# Positive autoantibodies to ZnT8 indicate elevated risk for additional autoimmune conditions in patients with Addison’s disease

**DOI:** 10.1007/s12020-016-0916-7

**Published:** 2016-03-14

**Authors:** Marta Fichna, Anita Rogowicz-Frontczak, Magdalena Żurawek, Piotr Fichna, Maria Gryczyńska, Dorota Zozulińska-Ziółkiewicz, Marek Ruchała

**Affiliations:** Department of Endocrinology, Metabolism and Internal Medicine, Poznan University of Medical Sciences, 49 Przybyszewskiego, 60-355 Poznan, Poland; Institute of Human Genetics, Polish Academy of Sciences, Poznan, Poland; Department of Internal Medicine and Diabetology, Poznan University of Medical Sciences, Poznan, Poland; Department of Paediatric Diabetes and Obesity, Poznan University of Medical Sciences, Poznan, Poland

**Keywords:** Addison’s disease, ZnT8 autoantibody, Diabetes, Autoimmunity

## Abstract

Autoimmune Addison’s disease (AAD) associates with exceptional susceptibility to develop other autoimmune conditions, including type 1 diabetes (T1D), marked by positive serum autoantibodies to insulin (IAA), glutamic acid decarboxylase (GADA) and insulinoma-associated protein 2 (IA-2A). Zinc transporter 8 (ZnT8) is a new T1D autoantigen, encoded by the *SLC30A8* gene. Its polymorphic variant rs13266634C/T seems associated with the occurrence of serum ZnT8 antibodies (ZnT8A). This study was designed to determine the prevalence of serum ZnT8A and their clinical implication in 140 AAD patients. Other beta cell and thyroid-specific autoantibodies were also investigated, and ZnT8A results were confronted with the rs13266634 genotype. ZnT8A were detectable in 8.5 %, GADA in 20.7 %, IA-2A in 5.7 %, IAA in 1.6 % and various anti-thyroid antibodies in 7.1–67.8 % individuals. Type 1 diabetes was found in 10 % AAD patients. ZnT8A were positive in 57.1 % of T1D patients and 3.4 % non-diabetic AAD. Analysis of ZnT8A enabled to identify autoimmunity in two (14.3 %) T1D individuals previously classified as autoantibody-negative. ZnT8A-positive patients revealed significantly higher number of autoimmune conditions (*p* < 0.001), increased prevalence of T1D (*p* < 0.001) and other beta cell-specific autoantibodies. Carriers of the rs13266634 T-allele displayed increased frequency (*p* = 0.006) and higher titres of ZnT8A (*p* = 0.002). Our study demonstrates high incidence of ZnT8A in AAD patients. ZnT8A are associated with coexisting T1D and predictive of T1D in non-diabetic subjects. Moreover, positive ZnT8A in AAD indicate elevated risk for additional autoimmune conditions. Autoantibodies to beta cell antigens, comprising ZnT8, could be included in routine screening panels in AAD.

## Introduction

Individuals suffering from the autoimmune Addison’s disease (AAD) display remarkable susceptibility to develop other autoimmune conditions, in particular organ-specific comorbidities, such as autoimmune thyroid disease (AITD), type 1 diabetes (T1D) and pernicious anaemia. Contemporary diagnostic tools enable early detection of concomitant autoimmune disorders in up to 80 % of AAD patients [1, 2]. Typical disease combinations are classified into distinct autoimmune polyendocrine syndromes (APS) and present significant challenge in clinical management of the AAD patients [3, 4]. The combination of AAD with T1D seems particularly disadvantageous. These patients may present with recurrent hypoglycaemic episodes, due to decreased gluconeogenesis and enhanced insulin sensitivity during glucocorticoid shortage [5]. On the other hand, conventional glucocorticoid replacement is non-physiological and may impair glycaemic control at times of exogenous steroid excess [6]. Registry-based data demonstrate elevated mortality rates among subjects with AAD and diabetes compared to non-diabetic AAD patients [7]. Therefore AAD individuals require close follow-up with regard to their diabetic risk. Both disorders share several similarities including predisposing genetic alleles and T cell-mediated destruction of the endocrine tissue. Additionally, AAD as well as T1D manifest with organ-specific autoantibodies, which are markers of an on-going autoimmune process detectable prior to clinical presentation. Positive serum autoantibodies to pancreatic islet antigens: insulin (IAA), glutamic acid decarboxylase (GADA) and insulinoma-associated protein 2 (IA-2A) are hallmarks of beta cell-directed autoimmunity, and were studied in the AAD cohorts in the past [1, 2, 8, 9]. On the contrary, recently discovered zinc transporter 8 autoantibodies (ZnT8A) have not been investigated in patients with AAD to date.

ZnT8 is a cation efflux transporter, localized in the membrane of the beta cell secretory granules, which is mandatory for insulin maturation and crystallization [10–12]. ZnT8 was identified as plausible beta cell autoantigen based on microarray transcript profiling of the islet cells, and ZnT8A were detected in sera from 60 to 80 % of Caucasian new-onset T1D patients [13]. Adding ZnT8A measurement to previously known serologic markers of diabetes enhanced autoimmunity detection rates to 98 % at disease onset [13]. Moreover, ZnT8A appeared as a useful predictive tool in the first-degree relatives of the T1D patients [14, 15]. Finally, ZnT8A evaluation proved practical in distinguishing non-autoimmune diabetes from the latent autoimmune diabetes in adults (LADA) where combined presence of GADA, IA-2A and ZnT8A is indicative of a more severe degree of insulin deficiency [16, 17].

ZnT8 is encoded by *SLC30A8* gene located on chromosome 8q24.11 [10]. Its polymorphic variant, rs13266634 C/T, which results in a missense R325W substitution, was identified as susceptibility factor for type 2 diabetes (T2D) however, functional mechanism underlying this association has not been elucidated [18]. Fluorescence-based experiments in cell lines suggest that W325 variant may encode a more active zinc transporter, whereas data from isolated human islets did not confirm improved insulin secretion [11, 19]. Homozygous CC offspring of T2D individuals displayed decreased first-phase insulin release in response to intravenous glucose load [20]. High-risk CC genotype was also connected with elevated proinsulin/insulin ratio in non-diabetic subjects [21]. Nevertheless, in contrast to the well-established link with T2D, no association between *SLC30A8* polymorphism and T1D was found and the locus has not been pinpointed in any genome-wide scan [22, 23]. However, rs1326634 appeared associated with the occurrence and with allele specificity of ZnT8A in T1D [14, 24].

Current study was designed to investigate serum autoantibodies to ZnT8 in a cohort of AAD patients and to evaluate them with regard to coexisting diabetes, other autoimmune conditions and the presence of additional serum autoantibodies. Additionally, we performed genotyping of rs13266634 in order to verify if *SLC30A8* variant might be associated with the occurrence of ZnT8A in this population.

## Materials and methods

### Patients

This cross-sectional analysis comprised 140 patients with AAD (101 females, 39 males) aged 47.5 ± 15.0 (range 18–92) years, admitted to the endocrine departments at the Poznan University of Medical Sciences between 2007 and 2014. The diagnosis of adrenal failure was established as previously described [25]. Autoimmune aetiology of the disease was confirmed by lack of infiltrative adrenal lesions in computed tomography scans and positive serum sampling for anti-adrenal autoantibodies. These antibodies had been formerly evaluated by an in-house solid-phase radioimmunoassay (RIA) using microsomal fraction of human adrenals and, in recently diagnosed individuals, by a commercial RIA assay (RSR Ltd, Cardiff, UK) which quantifies serum autoantibodies to 21-hydroxylase, the main adrenal autoantigen [26]. At the time of the study, all patients were receiving glucocorticoid replacement with hydrocortisone (HC) tablets and their mean daily HC dose was 24.4 ± 3.8 mg. Additionally, 117 (83.6 %) individuals were on fludrocortisone substitution (0.05–0.1 mg per day) and 27 (19.3 %) were taking dehydroepiandrosterone (12.5–25 mg daily). Medical records were revised in all patients in search for other previously diagnosed autoimmune conditions. Current complaints and clinical symptoms were carefully analysed with regard to new-onset autoimmunity, and additional laboratory workup was undertook if appropriate. The study was approved by the ethical committee at Poznan University of Medical Sciences and informed consent was obtained from all participants.

### Autoantibody measurements

Blood samples were taken in the morning, after overnight fast. Thyroid-specific autoantibodies were evaluated at once, as a part of regular check-up in AAD. BRAHMS (Henningsdorf, Germany) RIA assays were applied to test for antibodies to thyroid peroxidase (aTPO), thyroglobulin (aTg) and TSH receptor (TRAb) on a scintillation gamma counter (CliniGamma 1272, LKB Wallac, Finland). Autoantibody values of aTPO <60 U/mL, aTg <60 U/mL and TRAb <2 U/L, were considered negative, as advised by the manufacturer. Serum samples for assessment of beta cell-specific autoantibodies were collected and stored at −20 °C until analysed. Autoantibodies were evaluated using assays from RSR Ltd. (Cardiff, UK): RIAs for antibodies to GAD (cut-off >1.0 U/mL), IA-2 (cut-off >1.0 U/mL) and insulin (cut-off ≥0.4 U/mL), and ELISA for antibodies to ZnT8 C-terminal region. RSR’s ZnT8A assay is capable of detecting and quantifying autoantibodies specific to R325, W325 or residue 325 non-specific variants [27]. According to the manufacturer’s recommendation results <15 U/mL were considered negative. Patients with T1D on insulin treatment were excluded from IAA analysis as positive insulin antibodies could reflect genuine hallmark of autoimmunity as well as a treatment-related phenomenon, commonly found after initiation of exogenous insulin substitution.

### Biochemical analysis

Patients with positive beta cell-specific autoantibodies and no previous history of diabetes underwent standard (75 g) oral glucose tolerance test with simultaneous measurement of glucose and insulin levels (electrochemiluminescent method using Modular Analytics E170 and commercial assay from Roche Diagnostics). Haemoglobin A1c (HbA1c) was evaluated using Architect HbA1c Immunoassay (Abbott Diagnostics, IL, USA).

### Genotyping

Genomic DNA was extracted from the peripheral blood using Gentra Puregene Blood Kit (Qiagen, Hilden, Germany). Genotyping of the *SLC30A8* rs13266634 was performed by allelic discrimination analysis using the 7900HT Real-Time PCR System and TaqMan SNP Genotyping assay C_357888_10, following the conditions recommended by the manufacturer (Applied Biosystems, Foster City, CA). Data acquisition and analysis were performed using the allelic discrimination module in SDS v2.3 software (Applied Biosystems). The genotypes were confirmed in 5 % of samples by direct DNA sequencing with BigDye Terminator Cycle Sequencing Ready Reaction Kit (ABI Prism 3730 Genetic Analyzer, Foster City, CA) and 15 % randomly chosen samples were re-genotyped to ensure accuracy.

### Statistical analysis

Statistical calculations were performed by GraphPad Prism v.6.0 (La Jolla, CA, USA). Data are presented as mean ± standard deviation (±SD) unless stated otherwise. Their distribution was evaluated using the Kolmogorov–Smirnov test. Normally distributed data were compared using the unpaired Student t-test, and those with non-normal distribution were analysed by the non-parametric Mann–Whitney test. Statistical correlations were assessed by calculation of Pearson’s or Spearman’s coefficient, depending on data distribution. Categorical variables were compared using *χ*^2^ or Fisher’s exact test where appropriate. Two-tailed *p*-values <0.05 were considered significant.

Rs13266634 genotypes were checked for Hardy–Weinberg equilibrium (threshold *p* < 0.05) using online calculator available at the Helmholtz Center (Institut fur Humangenetik, Munich) website. Allele-wise and genotype-wise comparisons were tested on 2 × 2 and 3 × 2 contingency tables. Logistic regression analysis was applied to determine the odds ratios (ORs) and their 95 % confidence intervals (95 % CI).

## Results

The studied AAD group consisted of 140 subjects, whose mean age at disease onset was 36.3 ± 12.9 (range 9–69) years and mean disease duration 10.9 ± 10.3 (range 0–46) years (Table 1). Male patients were significantly younger compared to females, in line with their earlier AAD onset: 29.7 ± 13.1 versus 38.9 ± 11.9 years (*p* < 0.001). Only 21 (15 %) patients presented with isolated AAD, two siblings suffered from APS type 1, whereas all other participants displayed either APS type 2 or 4, with up to five coexisting autoimmune conditions [3]. The individual number of autoimmune diseases was neither associated with gender nor correlated with age (Table 1).

**Table 1:** Clinical characteristics of 140 adults with autoimmune Addison’s disease (AAD) and comparison between female and male patients

Addison’s patients	All (%)	Female (%)	Male (%)	*p* value
n (%)	140	101 (72.1)	39 (27.9)	NA
Age (years)	47.5 ± 15.0	50.9 ± 13.9	38.7 ± 14.1	**<0.001**
Age at AAD onset (years)	36.3 ± 12.9	38.9 ± 11.9	29.7 ± 13.1	**<0.001**
AAD duration (years)	10.9 ± 10.3	11.6 ± 10.9	8.9 ± 8.2	0.369
No of autoimmune conditions				
1 = AAD only (%)	21 (15.0)	12 (11.9)	9 (23.1)	0.096
2 (%)	78 (55.7)	56 (55.4)	22 (56.4)	0.918
3 (%)	28 (20.0)	22 (21.8)	6 (15.4)	0.396
4 (%)	10 (7.2)	9 (8.9)	1 (2.6)	0.450
5 (%)	3 (2.1)	2 (2.0)	1 (2.6)	1.000
T1D (%)	14 (10)	9 (8.9)	5 (12.8)	0.534
Age at T1D onset (years)	41.6 ± 15.8	48.3 ± 13.3	28.2 ± 9.0	**0.011**
T1D duration (years)	5.5 ± 3.8	6.1 ± 3.1	4.6 ± 5.0	0.384
AITD (%)	105 (75.0)	84 (83.2)	21 (53.8)	**<0.001** ^a^
Hashimoto’s thyroiditis (%)	81 (57.9)	64 (63.4)	17 (43.6)	**0.034** ^b^
Graves’ disease (%)	24 (17.1)	20 (19.8)	4 (10.3)	0.218
Chronic atrophic gastritis with or without pernicious anaemia (%)	21 (15.0)	14 (13.9)	7 (17.9)	0.544
Hypergonadotropic hypogonadism (%)	9 (6.4)	8 (7.9)	1 (2.6)	0.445
Hypoparathyroidism (%)	2 (1.4)	0	2 (5.1)	NA
Vitiligo (%)	8 (5.7)	6 (5.9)	2 (5.1)	1.000
Alopecia (%)	3 (2.1)	2 (2.0)	1 (2.6)	1.000

*p* values in bold indicate statistically significant results

^a^OR (95 % CI) 4.26 (1.870–9.593)

^b^OR (95 % CI) 2.24 (1.056–4.746)

Type 1 diabetes was found in 14 (10 %) of AAD patients, with comparable frequency among females and males (*p* = 0.534) (Table 1). T1D was diagnosed based on classical symptoms at the onset, blood glucose concentration >11.1 mmol/L and the presence of at least one beta cell-specific autoantibody. All except for one male presented with initial ketoacidosis. All patients defined as T1D cases required immediate treatment with insulin. Mean age at T1D onset was 41.6 ± 15.8 (range 16–68) years and mean T1D duration at the time of serum sampling was 5.5 ± 3.8 (range 0.5–13) years. The disease emerged earlier in male patients (*p* = 0.011), at an age similar to their AAD onset. AAD preceded T1D in eight individuals (in average by 11.3 ± 7.8 years), whereas T1D was diagnosed before adrenal failure in five patients (spaced by 6.6 ± 6.2 years), and both disorders appeared at the same time in one female. Details of the associated autoimmune conditions are displayed in Table 2. Additionally 9 (6.4 %) AAD patients had been previously diagnosed with T2D, based on their glucose levels in standard oral glucose tolerance test. They were treated with oral hypoglycaemic agents and diet.

**Table 2:** Detailed clinical characteristics of patients with autoimmune Addison’s disease (AAD) and concomitant type 1 diabetes (T1D)

	Sex	Age (years)	No of autoimmune conditions	Sequence of the autoimmune conditions with the age at their onset (years)
1	M	20	2	T1D (16), AAD (17)
2	F	29	3	HT (27), T1D (28), AAD (28)
3	M	32	3	GD (17), AAD (21), T1D (31)
4	M	33	3	T1D (16), HT (24), AAD (32)
5	F	38	3	AAD (29), HT (29), T1D (36)
6	M	43	4	AAD (39), sHT (39), ChG (41), T1D (42)
7	M	48	5	AAD (32), HT (39), ChG (40), HH (40), T1D (43)
8	F	50	4	HT (24), vitiligo (26), T1D (37), AAD (49)
9	F	54	3	AAD (39), HT (40), T1D (49)
10	F	56	4	AAD (34), HT (38), T1D (47), ChG (53)
11	F	59	4	POI (33), AAD (45), HT (45), T1D (49)
12	F	64	3	GD (34), T1D (57), AAD (59)
13	F	72	3	T1D (64), AAD (66), HT (67)
14	F	77	3	AAD (40), HT (55), T1D (68)

*HT* Hashimoto’s thyroiditis, *sHT* subclinical HT (positive serum autoantibodies and typical thyroid ultrasound with no alteration in thyroid function tests), *GD* Graves’ disease, *ChG* chronic gastritis, *HH* hypergonadotropic hypogonadism, *POI* premature ovarian insufficiency

Proportions of positive beta cell and thyroid autoantibodies are displayed in Table 3. ZnT8A were detectable in 8.5 %, GADA in 20.0 %, IA-2A in 5.7 %, IAA in 1.6 % (excluding subjects on insulin treatment), aTPO in 67.8 %, aTg in 45.0 %, and TRAb in 7.1 % of AAD individuals. Gender-related differences were found only for aTPO, which appeared more common in female patients [OR 2.35 (95 % CI 1.09–5.07) *p* = 0.027].

**Table 3:** Positive serum autoantibodies to beta cell and thyroid antigens found in autoimmune Addison’s disease (AAD)

Addison’s patients	All (%)	Female (%)	Male (%)	*p* value
*n* (%)	140	101 (72.1)	39 (27.9)	NA
ZnT8A	12 (8.5)	8 (7.9)	4 (10.3)	0.738
GADA	28 (20.0)	20 (19.8)	8 (20.5)	0.925
IA-2A	8 (5.7)	6 (5.9)	2 (5.1)	1.000
IAA^a^	2 (1.6)	2 (2.2)	0	NA
aTPO	95 (67.8)	74 (73.3)	21 (53.8)	**0.027**
aTg	63 (45.0)	42 (41.6)	21 (53.8)	0.191
TRAb	10 (7.1)	9 (8.9)	1 (2.6)	0.283

*p* values in bold indicate statistically significant results

^a^Proportion calculated with the exclusion of 14 patients with type 1 diabetes, already treated with insulin at the time of autoantibody investigation

**Fig. 1:**
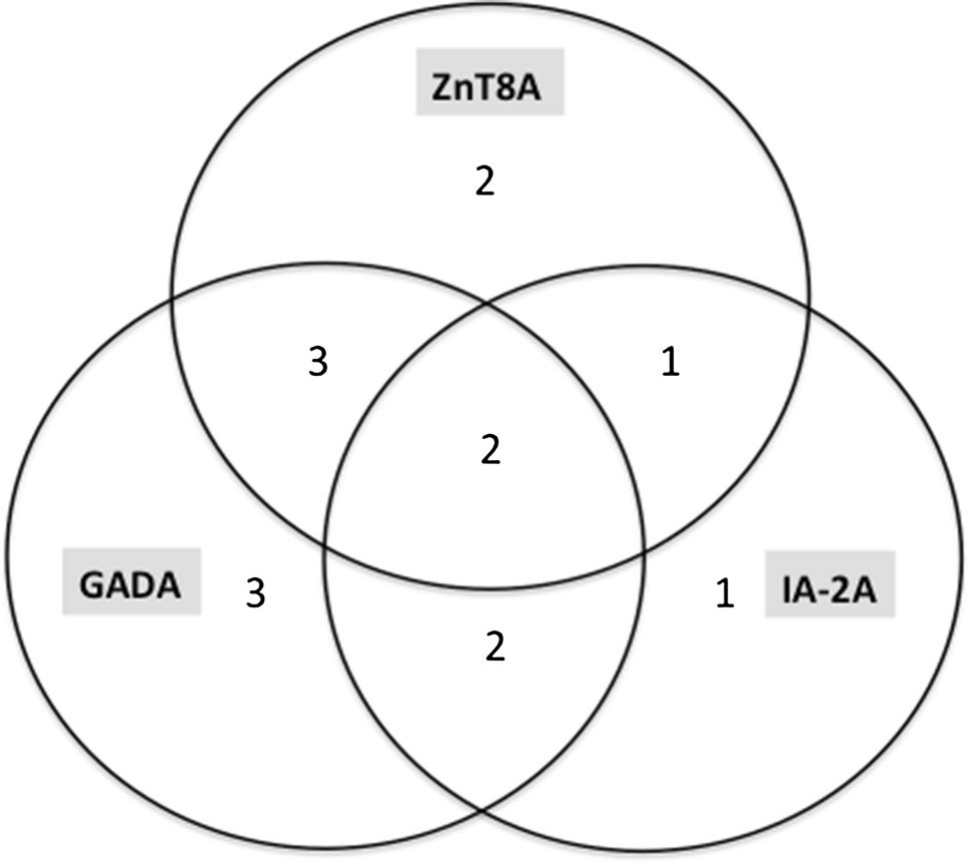
Co-occurrence of autoantibodies to zinc transporter 8 (ZnT8A), glutamic acid decarboxylase (GADA) and insulinoma-associated protein 2 (IA-2A) in patients with autoimmune Addison’s disease and concomitant type 1 diabetes

Positive serum autoantibodies to beta cell antigens were identified in 37 (26.4 %) patients: all individuals with T1D, two with T2D (22.2 %) and 21 (17.9 %) with no history of diabetes (Table 4). GADA remained the most common finding, detectable in 71.4 % T1D, 11.1 % T2D and 14.5 % non-diabetic AAD individuals. ZnT8A were found in 57.1 % of T1D patients and 3.4 % non-diabetic AAD, respectively. 42.9 % of T1D subjects presented with two positive autoantibody specificities and two T1D persons displayed reactivity to three beta cell antigens: ZnT8, IA-2 and GAD (Table 4). Figure 1 presents overlapping prevalence of beta cell autoantibodies in our AAD + T1D group (14 individuals). The inclusion of ZnT8A provided evidence for autoimmune origin of diabetes in two (14.3 %) T1D patients who otherwise would be classified as autoantibody-negative. Combined analysis of three autoantibody specificities allowed identification of beta cell autoimmunity in all T1D individuals in this series.

**Table 4:** Frequency of serum autoantibodies to beta cell antigens in patients with autoimmune Addison’s disease (AAD)

Positive serum autoantibody	All AAD *n* = 140 (%)	AAD + T1D *n* = 14 (%)	AAD + T2D *n* = 9 (%)	AAD nonDM *n* = 117 (%)
ZnT8A	12 (8.5)	8 (57.1)	0	4 (3.4)
GADA	28 (20.7)	10 (71.4)	1 (11.1)	17 (14.5)
IA-2A	8 (5.7)	6 (42.8)	0	2 (1.7)
IAA^a^	2 (1.6)	NA	1 (11.1)	1 (0.9)
1 autoantibody	26 (18.6)	6 (42.9)	2 (22.2)	18 (15.4)
2 autoantibodies	9 (6.4)	6 (42.9)	0	3 (2.6)
3 autoantibodies	2 (1.4)	2 (14.2)	0	0
Total	37 (26.4)	14 (100.0)	2 (22.2)	21 (17.9)

Positive autoantibody results are further classified according to diabetic status: with type 1 diabetes (AAD + T1D), with type 2 diabetes (AAD + T2D) or without history of diabetes (AAD nonDM)

^a^Proportion calculated with the exclusion of 14 patients with type 1 diabetes i.e. already treated with insulin at the time of autoantibody investigation

Single positive autoantibodies were also detected in 2 (22.2 %) AAD + T2D patients. Moreover, positive serologic markers of beta cell autoimmunity were found in 21 (17.9 %) AAD individuals with no previous history of diabetes (Table 4), comprising three females who displayed reactivity to two beta cell antigens (GADA + ZnT8A, GADA + IAA and ZnT8 + IA-2A). Further biochemical assessment in the autoantibody-positive non-diabetic subgroup revealed mean fasting plasma glucose and insulin 4.8 ± 0.6 mmol/L and 8.5 ± 3.4 μU/mL, respectively. Standard oral glucose challenge showed respective mean 2 h glucose and insulin levels of 8.3 ± 1.2 mmol/L and 57.1 ± 16.8 μU/mL, providing evidence of glucose tolerance in three individuals. Mean HbA1c in non-diabetic beta cell autoantibody-positive AAD patients was 5.5 ± 0.4 %.

Stratification of the AAD cohort by serum ZnT8A results revealed significantly higher number of autoimmune conditions in ZnT8A-positive subjects (*p* < 0.001) (Table 5). As expected, those individuals displayed increased prevalence of T1D compared to ZnT8A-negative patients (*p* < 0.001). Other beta cell-specific autoantibodies were also more frequent in subjects reactive to ZnT8A (Table 5). No association of ZnT8A with markers of thyroid autoimmunity was detected (all *p* > 0.05).

**Table 5:** Comparison of clinical and serological findings between ZnT8 autoantibody (ZnT8A)-positive and -negative patients with autoimmune Addison’s disease

	ZnT8A-positive (%)	ZnT8A-negative (%)	*p* value
*n*	12	128	
Sex	8 F (66.7)	93 F (72.7)	0.738
Age (years)	49.3 ± 14.5	47.2 ± 15.1	0.657
Age at AAD onset (years)	38.0 ± 11.5	35.9 ± 12.9	0.596
AAD duration (years)	11.2 ± 8.7	10.9 ± 10.5	0.690
No. of autoimmune disorders	3.3 ± 1.1	2.2 ± 0.8	**<0.001**
T1D co-occurrence (%)	8 (66.7)	6 (4.7)	**<0.001** ^a^
Age at T1D onset (years)	38.5 ± 15.0	46.2 ± 17.0	0.374
T1D duration (years)	5.4 ± 3.3	6.2 ± 4.8	0.739
ZnT8A titre (U/mL)	29.4 (19.2–167.3)	6.9 (6.7–8.6)	**<0.001**
GADA positive (%)	6 (50.0)	22 (17.2)	**0.007** ^b^
GADA titre (U/mL)	18.1 (0.2–62.4)	0.2 (0–0.4)	**0.001**
IA-2A positive (%)	4 (33.3)	4 (3.1)	**0.002** ^c^
IA-2A titre (U/mL)	0.5 (0.1–2.5)	0.1 (0–0.3)	**0.002**
IAA positive (%)	0	2 (1.6)	NA
TSH (mIU/L)	2.25 (1.31–4.25)	2.20 (1.10–3.40)	0.406
AITD (%)	11 (91.7)	94 (73.4)	0.294
Hashimoto’s thyroiditis (%)	10 (83.3)	71 (55.5)	0.073
Graves’ disease (%)	1 (8.3)	23 (17.9)	0.691
aTPO positive (%)	9 (75.0)	86 (67.2)	0.579
aTPO titre (U/mL)	242 (94–558)	245 (46–976)	0.749
aTg positive (%)	8 (66.7)	55 (43.0)	0.137
aTg titre (U/mL)	105 (42–556)	50 (19–150)	0.099
TRAb positive (%)	1 (8.3)	9 (7.0)	1.000
TRAb titre (mIU/L)	0.7 (0.3–1.1)	0.5 (0.2–1.0)	0.363

*p* values in bold indicate statistically significant results

Normally distributed values are displayed as mean ± standard deviation, whereas those with non-Gaussian distribution are presented as median (25th–75th centile)

^a^OR (95 % CI) 40.70 (9.50–174.0)

^b^OR (95 % CI) 4.82 (1.42–16.34)

^c^OR (95 % CI) 15.5 (3.26–73.40)

Genotyping of the *SLC30A8* variant rs13266634 revealed 66 (47.1 %) CC homozygotes, 62 (44.3 %) heterozygotes and 12 (8.6 %) mutant TT homozygotes in our AAD cohort, which remained in Hardy–Weinberg equilibrium (*p* = 0.631). ZnT8A were positive in 6.0 % of CC, 6.5 % of CT and 33.3 % of TT individuals, respectively (*p* = 0.006). In accordance, the latter subgroup displayed the highest titres of circulating ZnT8A compared to CT and CC subjects (with medians 10.7, 8.2 and 6.8 U/mL, respectively; *p* = 0.002). The presence of the T-allele was associated with increased risk of developing ZnT8A in AAD [14.0 vs. 6.2 %, OR 2.46 (95 % CI 1.06–5.72) *p* = 0.032].

## Discussion

Our study provides the first systematic evaluation of ZnT8A in a large, clinically well-defined AAD cohort. 85 % AAD individuals presented with at least one additional disorder, which confirms their exceptional susceptibility to develop other autoimmune conditions [2, 8, 9, 28, 29]. Neither age nor sex seemed to influence the individual number of associated diseases [8, 30]. Nonetheless, thyroid autoimmunity, and Hashimoto’s thyroiditis in particular, was more prevalent in AAD females than males. In contrast, no gender difference was found for T1D, diagnosed in 10 % of patients. Previous studies revealed variable incidence of T1D among AAD patients, ranging from 1.2 to 20.4 % [2, 8, 9, 28–30]. Different methods of data collection, variable degree of stringency in selection of patients with autoimmune adrenal failure and population differences might account for this discrepancy. Most recent analyses in European AAD cohorts report 10–15 % of T1D cases [2, 8, 29].

In our study AAD was diagnosed prior to T1D in 57.1 % of patients. In most formerly described cases AAD followed the diagnosis of T1D, although all possible patterns have been reported [2, 8, 9, 28–30]. Relatively old mean age at T1D onset, exceeding 35 years in 10 out of 14 affected AAD patients, could suggest LADA. However, most of them presented with initial ketoacidosis and all required immediate insulin therapy, which supports remarkable intensity of the auto-aggressive process. All AAD + T1D patients, except for the youngest person, displayed additional autoimmune conditions, most commonly thyroid disorders, resulting in a classical combination of AAD, T1D and thyroid autoimmunity (Carpenter’s syndrome) [3].

Antibodies typical for beta cell autoaggression were found in a considerable proportion of our AAD patients. Former studies, based on indirect immunofluorescence assays using cryostatic sections of human pancreas, confirmed increased prevalence of islet cell autoantibodies in AAD. A few reports addressed specifically the prevalence of GADA and IA-2A (detectable in 16.0–21.0 and 8.5–11.0 % of AAD cases, respectively) [1, 2, 8, 9, 30]. Preliminary investigation of sera from 35 AAD subjects conducted upon initial ZnT8A description, revealed 8.6 % ZnT8A positivity, but no clinical characteristic of those patients was available [13]. Our analysis found the same frequency of ZnT8A (8.5 %) with no gender differences. In line with other studies in AAD, GADA were most prevalent in subjects with concomitant T1D (71.4 %) [1, 9, 30]. According to our results ZnT8A appeared as the second most frequent (57.1 %) marker of pancreatic autoimmunity in T1D among AAD individuals. GADA and ZnT8A together were the most common combination of beta cell autoantibodies (Fig. 1). Adding ZnT8A allowed to identify autoimmunity in two T1D patients, who based solely upon measurements of previously known markers would be classified as autoantibody-negative diabetes. Extending the classical panel of diabetes-associated autoantibodies with ZnT8A enables to increase its positive predictive value for T1D or LADA [13, 16, 17].

Autoantibodies to beta cell antigens were also found in two AAD + T2D patients, challenging their formerly established diagnosis. Current results suggest that these patients could be re-classified as LADA cases. Positive beta cell-specific autoantibodies are sometimes found in phenotypically defined T2D cases [31]. Nonetheless, very low incidence of ZnT8A positivity was reported in T2D, which remains in line with the negative result in our cohort [13, 16].

Finally, a notable proportion of non-diabetic AAD patients displayed positive beta cell autoantibodies. Analysis of ZnT8A enabled detection of two autoantibody-positive subjects and provided additional autoimmune marker in two others. Double autoantibody reactivity was detected in three individuals without previously known glycaemic disorders. Upon subsequent investigation, three of non-diabetic patients, including one with double autoantibody positivity, presented impaired glucose tolerance, indicating high risk of diabetes and the need of close follow-up [14, 15, 32].

ZnT8A-positive AAD patients presented increased susceptibility to develop additional autoimmune conditions with significantly higher number of concomitant diseases. Subjects who were positive for ZnT8A displayed predictably higher incidence of T1D with increased frequencies and titres of other beta cell autoantibodies [13, 17, 32]. Our former analysis in newly diagnosed LADA patients suggested that positive ZnT8A might predict increased occurrence of serum aTPO [17]. Both GADA and ZnT8A were also associated with autoimmune thyroid disease in T2D [33]. Therefore, ZnT8A could be a marker of a more severe phenotype of organ-specific autoimmunity. However, in accordance with the observations conducted in children with T1D, our data do not support specific link between ZnT8A and thyroid autoimmunity [27]. This apparent discrepancy may arise from different timings of ZnT8A measurements after T1D diagnosis. In most instances ZnT8A tend to decrease rapidly after diabetes onset, although they are still present in approximately 40 % of T1D patients 5 years post diagnosis [27, 34].

Genotyping of *SLC30A8* polymorphism in our AAD group revealed genotype distribution similar to the large control cohorts of European origin [20, 22]. As previously described in T1D, rs13266634 TT homozygotes displayed increased proportion and higher titres of serum ZnT8A [23]. *SLC30A8* polymorphism at the codon 325 results in the expression of three protein variants: R325, W325 (rs13266634) and very rarely Q325, if the nucleotide substitution is located in the second nucleotide of this codon (rs16889462). ZnT8A may interact with the R325 or W325 variant, or may be residue 325 non-specific whereas sera that react uniquely with the Q325 allele are extremely rare [13, 24]. Remarkably, ZnT8A from individuals homozygous for the *SLC30A8* genotypes are often limited to their respective ZnT8 isoform [14, 24]. Individuals heterozygous for rs13266634 seem to display the lowest incidence of ZnT8A but we could not confirm this observation in our cohort [14, 23].

This study is the first comprehensive description of serum ZnT8A in an AAD cohort however it presents some limitations. First of all, sample size is moderate due to low AAD incidence [2, 4]. However, its strength relies on very well-documented individual data from each patient, excluding risk of any misclassification, possible in the registry-based studies. Another issue is the cross-sectional character of our analysis, which was conducted in individuals of variable disease duration, ranging from just a few weeks up to several years. Some antibodies, including ZnT8A, might have vanished by the time of the study in a proportion of patients. Systematic evaluation of a wide array of autoantibodies at AAD onset and subsequently at fixed intervals might shed more light on dynamics of the autoimmune process. Eventually, technical limitations of the ZnT8A assay might play some role. We used a commercial ELISA kit, which employs a dimer of ZnT8 C-terminal domain (amino acids 275–369) containing both R325 and W325 alleles, but does not distinguish Q325 specificity [27]. It has been validated in Islet Autoantibody Standardization Program 2012 and 2013, yielding high sensitivity and specificity scores [27]. Some reports suggested that analysing all three ZnT8A variants might improve the diagnostic sensitivity of T1D, however, only 0.73 % of affected children were positive for Q325-specific ZnT8A [35].

In conclusion, our study demonstrates high incidence of ZnT8A in AAD patients. ZnT8A are associated with coexisting T1D and, together with GADA, may point towards the risk of glycaemic disorders in non-diabetic AAD subjects. Moreover, positive ZnT8A among AAD individuals indicate particularly elevated risk for additional autoimmune conditions. Considering the frequent occurrence of T1D in AAD cohorts, serum autoantibodies to beta cell antigens, comprising ZnT8, should be included in the regular screening panels in this high-risk population.

## References

[1] Fichna M, Fichna P, Gryczynska M, Walkowiak J, Zurawek M, Sowinski J (2010). Screening for associated autoimmune disorders in Polish patients with Addison’s disease. Endocrine.

[2] Betterle C, Scarpa R, Garelli S, Morlin L, Lazzarotto F, Presotto F, Coco G, Masiero S, Parolo A, Albergoni MP, Favero R, Barollo S, Salve M, Basso D, Chen S, Rees Smith B, Furmaniak J, Mantero F (2013). Addison’s disease: a survey on 633 patients in Padova. Eur. J. Endocrinol..

[3] Neufeld M, Maclaren N, Blizzard R (1980). Autoimmune polyglandular syndromes. Pediatr. Ann..

[4] Husebye ES, Allolio B, Arlt W, Badenhoop K, Bensing S, Betterle C, Falorni A, Gan EH, Hulting AL, Kasperlik-Załuska A, Kampe O, Lovas K, Meyer G, Pearce SH (2014). Consensus statement on the diagnosis, treatment and follow-up of patients with primary adrenal insufficiency. J. Int. Med..

[5] McAulay V, Frier BM (2000). Addison’s disease in type 1 diabetes presenting with recurrent hypoglycaemia. Postgrad. Med. J..

[6] Elbelt U, Hahner S, Allolio B (2009). Altered insulin requirement in patients with type 1 diabetes and primary adrenal insufficiency receiving standard glucocorticoid replacement therapy. Eur. J. Endocrinol..

[7] Bergthorsdottir R, Leonsson-Zachrisson M, Oden A, Johannsson G (2006). Premature mortality in patients with Addison’s disease: a population-based study. J. Clin. Endocrinol. Metab..

[8] Erichsen MM, Lovas K, Skinningsrud B, Wolff AB, Undlien DE, Svartberg J, Fougner KJ, Berg TJ, Bollerslev J, Mella B, Carlson JA, Erlich H, Husebye ES (2009). Clinical, immunological, and genetic features of autoimmune primary adrenal insufficiency: observations from a Norwegian registry. J. Clin. Endocrinol. Metab..

[9] Ross I, Boulle A, Soule S, Levitt N, Pirie F, Karlsson A, Mienie J, Yang P, Wang H, She JX, Winter W, Schatz D (2010). Autoimmunity predominates in a large South African cohort with Addison’s disease of mainly European descent despite long-standing disease and is associated with HLA DQB*0201. Clin. Endocrinol. (Oxf.).

[10] Chimienti F, Devergnas S, Favier A, Seve M (2004). Identification and cloning of a beta-cell-specific zinc transporter, ZnT-8, localized into insulin secretory granules. Diabetes.

[11] Nicolson TJ, Bellomo EA, Wijesekara N, Loder MK, Baldwin JM, Gyulkhandanyan AV, Koshkin V, Tarasov AI, Carzaniga R, Kronenberger K, Taneja TK, da Silva Xavier G, Libert S, Froguel P, Scharfmann R, Stetyuk V, Ravassard P, Parker H, Gribble FM, Reimann F, Sladek R, Hughes SJ, Johnson PR, Masseboeuf M, Burcelin R, Baldwin SA, Liu M, Lara-Lemus R, Arvan P, Schuit FC, Wheeler MB, Chimienti F, Rutter GA (2009). Insulin storage and glucose homeostasis in mice null for the granule zinc transporter ZnT8 and studies of the type 2 diabetes-associated variants. Diabetes.

[12] Li YV (2014). Zinc and insulin in pancreatic beta-cells. Endocrine.

[13] Wenzlau JM, Juhl K, Yu K, Moua O, Sarkar SA, Gottlieb P, Pewers M, Eisenbarth GS, Jensen J, Davidson HW, Hutton JC (2007). The cation efflux transporter ZnT8 (Slc30A8) is a major autoantigen in human type 1 diabetes. Proc. Natl. Acad. Sci. USA.

[14] Achenbach P, Lampasona V, Landherr U, Koczwara K, Krause S, Grallert H, Winkler C, Pfluger M, Illig T, Bonifacio E, Ziegler AG (2009). Autoantibodies to zinc transporter 8 and SLC30A8 genotype stratify type 1 diabetes risk. Diabetologia.

[15] Gorus FK, Balti EV, Vermeulen I, Demeester S, Van Dalem A, Costa O, Dorchy H, Tenoutase S, Mouraux T, De Block C, Gillard P, Decochez K, Wenzlau JM, Hutton JC, Pipeleers DG, Weets I (2013). Belgian Diabetes Registry, Screening for insulinoma antigen 2 and zinc transporter 8 autoantibodies: a cost-effective and age-independent strategy to identify rapid progressors to clinical onset among relatives of type 1 diabetic patients. Clin. Exp. Immunol..

[16] Lampasona V, Petrone A, Tiberti C, Capizzi M, Spoletini M, di Pietro S, Songini M, Bonicchio S, Giorgino F, Bonifacio E, Bossi E, Buzzetti R, Non Insulin Requiring Autoimmune Diabetes (NIRAD) Study Group (2010). Zinc transporter 8 antibodies complement GAD and IA-2 antibodies in the identification and characterization of adult-onset autoimmune diabetes: non insulin requiring autoimmune diabetes (NIRAD) 4. Diabetes Care.

[17] Rogowicz-Frontczak A, Zozulinska-Ziolkiewicz D, Litwinowicz M, Niedzwiecki P, Wyka K, Wierusz-Wysocka B (2014). Are zinc transporter type 8 antibodies a marker of autoimmune thyroiditis in non-obese adults with new-onset diabetes?. Eur. J. Endocrinol..

[18] Sladek R, Rocheleau G, Rung J, Dina C, Shen L, Serre D, Boutin P, Vincent D, Belisle A, Hadjadj S, Balkau B, Heude B, Charpentier G, Hudson TJ, Montpetit A, Pshezhetsky AV, Prentki M, Posner BI, Balding DJ, Meyre D, Polychronakos C, Froguel P (2007). A genome-wide association study identifies novel risk loci for type 2 diabetes. Nature.

[19] Cauchi S, Del Guerra S, Choquet H, D’Aleo V, Groves CJ, Lupi R, McCarthy MI, Froguel P, Marchetti P (2010). Meta-analysis and functional effects of the SLC30A8 rs13266634 polymorphism on isolated human pancreatic islets. Mol. Genet. Metab..

[20] Boesgaard TW, Zilinskaite J, Vanttinen M, Laakso M, Jansson PA, Hammarstedt A, Smith U, Stefan N, Fritsche A, Haring H, Hribal M, Sesti G, Zobel DP, Pedersen O, Hansen T, EUGENE2 Consortium (2008). The common SLC30A8 Arg325Trp variant is associated with reduced first-phase insulin release in 846 non-diabetic offspring of type 2 diabetes patients—the EUGENE2 Study. Diabetologia.

[21] Kirchhoff K, Machicao F, Haupt A, Schafer SA, Tschritter O, Staiger H, Stefan N, Haring HU, Fritsche A (2008). Polymorphisms in the TCF7L2, CDKAL1 and SLC30A8 genes are associated with impaired proinsulin conversion. Diabetologia.

[22] Brorsson C, Bergholdt R, Sjogren M, Eising S, Sorensen KM, Hougaard DM, Orho-Melander M, Groop L, Pociot F (2008). A non-synonymous variant in SLC30A8 is not associated with type 1 diabetes in the Danish population. Mol. Genet. Metab..

[23] Howson JM, Krause S, Stevens H, Smyth DJ, Wenzlau JM, Bonifacio E, Hutton J, Ziegler AG, Todd JA, Achenbach P (2012). Genetic association of zinc transporter 8 (ZnT8) autoantibodies in type 1 diabetes cases. Diabetologia.

[24] Wenzlau JM, Liu Y, Yu L, Moua O, Fowler KT, Rangasamy S, Walters J, Eisenbarth GS, Davidson HW, Hutton JC (2008). A common nonsynonymous single nucleotide polymorphism in the SLC30A8 gene determines ZnT8 autoantibody specificity in type 1 diabetes. Diabetes.

[25] Fichna M, Fichna P, Gryczynska M, Czarnywojtek A, Zurawek M, Ruchala M (2015). Steroid replacement in primary adrenal failure does not appear to affect circulating adipokines. Endocrine.

[26] Kosowicz J, Gryczynska M, Bottazzo GF (1986). A radioimmunoassay for the detection of adrenal autoantibodies. Clin. Exp. Immunol..

[27] Petruzelkova L, Ananieva-Jordanova R, Vcelakova J, Vesely Z, Stechova K, Lebl J, Dusatkova P, Sumnik Z, Coles R, Powell M, Furmaniak J, Rees Smith B, Kolouskova S (2014). The dynamic changes of zinc transporter 8 autoantibodies in Czech children from the onset of Type 1 diabetes mellitus. Diabet. Med..

[28] Zelissen PM, Bast EJ, Croughs RJ (1995). Associated autoimmunity in Addison’s disease. J. Autoimmun..

[29] Leelarathna L, Breen L, Powrie JK, Thomas SM, Guzder R, McGowan B, Carroll PV (2010). Co-morbidities, management and clinical outcome of auto-immune Addison’s disease. Endocrine.

[30] Laureti S, Aubourg P, Calcinaro F, Rocchiccioli F, Casucci G, Angeletti G, Brunetti P, Lernmark A, Santeusanio F, Falorni A (1998). Etiological diagnosis of primary adrenal insufficiency using an original flowchart of immune and biochemical markers. J. Clin. Endocrinol. Metab..

[31] Turner R, Stratton I, Horton V, Manley S, Zimmet P, Mackay IR, Shattosck M, Bottazzo GF, Holman R (1997). UKPDS 25: autoantibodies to islet-cell cytoplasm and glutamic acid decarboxylase for prediction of insulin requirement in type 2 diabetes. UK Prospective Diabetes Study Group. Lancet.

[32] Yu L, Boulware DC, Beam CA, Hutton JC, Wnezlau JM, Greenbaum CJ, Bingley PJ, Krischer JP, Sosenko JM, Skyler JS, EIsenbarth GS, Mahon JL, Type 1 Diabetes TrialNet Study Group (2012). Zinc transporter-8 autoantibodies improve prediction of type 1 diabetes in relatives positive for the standard biochemical autoantibodies. Diabetes Care.

[33] Haller-Kikkatalo K, Pruul K, Kisand K, Nemvalts V, Reimand K, Uibo R (2015). R, GADA and anti-ZnT8 complicate the outcome of phenotypic type 2 diabetes of adults. Eur. J. Clin. Investig..

[34] Vaziri-Sani F, Oak S, Radtke J, Lernmark A, Lynch K, Agardh CD, Cilio CM, Lethagen AL, Ortqvist E, Landin-Olsson M, Torn C, Hampe CS (2010). ZnT8 autoantibody titers in type 1 diabetes patients decline rapidly after clinical onset. Autoimmunity.

[35] Andersson C, Larsson K, Vaziri-Sani F, Lynch K, Carlsson A, Cedervall E, Jonsson B, Neiderud J, Mansson M, Nilsson A, Lernmark A, Elding H (2011). Larsson, S.A. Ivarsson, The three ZNT8 autoantibody variants together improve the diagnostic sensitivity of childhood and adolescent type 1 diabetes. Autoimmunity.

